# Abstract of Meteorological Observations for June, 1855, Made at Philadelphia, Pa.

**Published:** 1855-08

**Authors:** James A. Kirkpatrick


*Abstract of Meteorological Observations for June,* 1855, *made at Philadelphia, Pa. Latitude* 39° 57′ 28″ *N., Longitude* 75° 10′ 40″ *W. from Greenwich.* By PROF. JAMES A. KIRKPATRICK.

| 1855. June. | Barometer. Daily Mean | Barometer. Mean Daily Range. | Thermom. Daily Mean | Thermom. Mean Daily Range | Dew Point 2 P.M. | Force of Vapor 2 P.M. | Rel. Humid. 2 P.M. | Rain. | Prevailing Winds. | Remarks. |
|---|---|---|---|---|---|---|---|---|---|---|
| | Inches. | Inches. | Deg. | Deg. | Deg. | Inches. | Hunds. | Inch. | Points. | |
| 1 | 29.809 | .235 | 70.3 | 3.2 | 62.6 | .568 | .67 | 1.600 | SSE. | *Rain* all day. |
| 2 | 29.657 | .152 | 66.3 | 4.0 | 64.8 | .613 | .90 | | SSE. | *Rain* all day. [with th'der. |
| 3 | 29.610 | .047 | 66.7 | 3.7 | 62.6 | .568 | .67 | 1.237 | SE. | Cl'y; aft. & night heavy *Rain* |
| 4 | 29.659 | .049 | 62.2 | 4.5 | 50.1 | .362 | .55 | | W. | M. and aft. cloudy; ev. clear. |
| 5 | 29.751 | .092 | 65.0 | 2.8 | 47.0 | .322 | .44 | | (Var.) | Clear. [and lightning. |
| 6 | 29.826 | .074 | 68.2 | 3.2 | 46.7 | .319 | .36 | 1.160 | SW. | Cl'y; *Rain* at n't, with th'der |
| 7 | 29.493 | .332 | 72.2 | 4.0 | 67.6 | .675 | .60 | 0.456 | SW. | M. *R'n*; aft. cl'y; ev. *R'n*, high wind, th'r. *Bar. l'st* 29.395. |
| 8 | 29.591 | .176 | 66.0 | 6.2 | 49.9 | .359 | .45 | | W. | Aft. cloudy; m. and ev. clear. |
| 9 | 29.681 | .090 | 68.7 | 2.7 | 49.5 | .354 | .42 | | (Var.) | M. cl'r; aft. & ev. cl'dy. [l'ng. |
| 10 | 29.469 | .212 | 72.3 | 7.0 | 64.0 | .597 | .53 | 0.586 | (Var.) | Cl'y; *R.* all n't; 7 to 8 P.M. th. & |
| 11 | 29.571 | .124 | 68.3 | 8.0 | 49.2 | .350 | .45 | 0.351 | W. | Cloudy; *rain* during night. |
| 12 | 29.810 | .239 | 64.3 | 4.0 | 41.3 | .260 | .38 | | NW. | Clear. |
| 13 | 29.945 | .138 | 64.3 | 3.3 | 47.0 | .322 | .44 | | NW. | Clear. *Th. lowest*, 51°. |
| 14 | 29.822 | .122 | 68.8 | 4.5 | 45.6 | .306 | .34 | | WNW. | Cloudy. |
| 15 | 29.657 | .166 | 72.5 | 3.5 | 50.1 | .362 | .37 | | W. | Cloudy. |
| 16 | 29.634 | .022 | 74.3 | 1.8 | 61.0 | .537 | .55 | 0.139 | (Var.) | Cl'y; ev. & n't *rain*, th. & l'ng. |
| 17 | 29.772 | .164 | 69.7 | 6.5 | 51.9 | .387 | .58 | 0.682 | NE. | Cloudy; m. *rain*. |
| 18 | 29.943 | .171 | 66.3 | 2.5 | 48.0 | .335 | .47 | | ESE. | Cloudy. |
| 19 | 29.833 | .114 | 60.0 | 6.3 | 56.4 | .455 | .88 | 0.220 | NE. | *Rain* all day. |
| 20 | 29.696 | .134 | 69.0 | 9.0 | 65.0 | .618 | .76 | | (Var.) | M. and aft. cloudy; ev. clear. |
| 21 | 29.742 | .046 | 75.7 | 6.7 | 63.4 | .585 | .56 | | (Var.) | Clear. |
| 22 | 29.644 | .098 | 75.0 | 2.0 | 73.1 | .813 | .82 | 0.546 | SSW. | Cloudy; aft. *rain*, th. & l'ng. |
| 23 | 29.806 | .162 | 77.7 | 2.7 | 67.3 | .668 | .61 | | NW. | Clear. [th'r & lightning. |
| 24 | 29.723 | .082 | 74.5 | 3.2 | 73.6 | .828 | .78 | 0.478 | (Var.) | Cloudy. M. *rain*; night rain, |
| 25 | 29.715 | .068 | 76.3 | 4.5 | 69.3 | .716 | .77 | 0.553 | SW. | M. *rain*; cloudy. |
| 26 | 29.869 | .155 | 79.7 | 4.7 | 66.4 | .648 | .59 | | SW. | M. and aft. cloudy; ev. clear. |
| 27 | 29.957 | .113 | 81.7 | 3.3 | 63.2 | .580 | .45 | | WSW. | Clear. *Bar. hig'st* 30.020. |
| 28 | 29.886 | .071 | 83.3 | 1.7 | 73.4 | .821 | .62 | | (Var.) | M. and aft. cloudy; ev. clear. |
| 29 | 29.833 | .052 | 88.0 | 4.7 | 73.7 | .831 | .51 | | (Var.) | Aft. cloudy; m. and ev. clear. |
| 30 | 29.774 | .059 | 88.7 | 1.3 | 77.5 | .941 | .61 | | (Var.) | M. cl'r; a. & e. cl'y; *Th. h'st* 95° |
| Means for June, 1855 | 29.739 | .125 | 71.9 | 4.2 | 59.4 | .537 | .57 | 8.008 | S. 76° W., 46–100. | |
| 4 yrs | 29.849 | .103 | 72.2 | 4.3 | 54.8 | | | 4.250 | S. 72° W., 46–100. | |

The Monthly Range of the Barometer was 0.625 of an inch., and of the Thermometer 44°.

